# Removal of natural anti-αGal antibodies elicits protective immunity against Gram-negative bacterial infections

**DOI:** 10.3389/fimmu.2023.1232924

**Published:** 2023-08-18

**Authors:** Sara Olivera-Ardid, Daniel Bello-Gil, Magdiel Perez-Cruz, Cristina Costa, Mariana Camoez, M. Angeles Dominguez, Yara Ferrero-Alves, Jose Miguel Vaquero, Nailya Khasbiullina, Nadezhda V. Shilova, Nicolai V. Bovin, Rafael Mañez

**Affiliations:** ^1^Infectious Pathology and Transplantation Division, Bellvitge Biomedical Research Institute (IDIBELL), Hospitalet de Llobregat, Spain; ^2^Microbiology Department, Bellvitge University Hospital, University of Barcelona, Hospitalet de Llobregat, Spain; ^3^Flow Cytometry Platform, Bellvitge Biomedical Research Institute (IDIBELL), Hospitalet de Llobregat, Spain; ^4^Shemyakin-Ovchinnikov Institute of Bioorganic Chemistry, Russian Academy of Sciences, Moscow, Russia; ^5^Intensive Care Department, Bellvitge University Hospital, Hospitalet de Llobregat, Spain

**Keywords:** antibody-dependent-enhancement of infection, anti-αGal antibodies, removal of antibodies, Gram-negative bacteria, protective immunity

## Abstract

Antibody-dependent enhancement (ADE) of bacterial infections occurs when blocking or inhibitory antibodies facilitate the infectivity of pathogens. In humans, antibodies involved in ADE of bacterial infections may include those naturally produced against Galα1-3Galβ1-4GlcNAcβ (αGal). Here, we investigate whether eliminating circulating anti-αGal antibodies using a soluble αGal glycopolymer confers protection against Gram-negative bacterial infections. We demonstrated that the *in vivo* intra-corporeal removal of anti-αGal antibodies in α1,3-galactosyltransferase knockout (GalT-KO) mice was associated with protection against mortality from Gram-negative sepsis after cecal ligation and puncture (CLP). The improved survival of GalT-KO mice was associated with an increased killing capacity of serum against *Escherichia coli* isolated after CLP and reduced binding of IgG1 and IgG3 to the bacteria. Additionally, inhibition of anti-αGal antibodies from human serum *in vitro* increases the bactericidal killing of *E. coli* O86:B7 and multidrug-resistant *Klebsiella pneumoniae* and *Pseudomonas aeruginosa.* In the case of *E. coli* O86:B7, there was also an improvement in bacteria opsonophagocytosis by macrophages. Both lytic mechanisms were related to a decreased binding of IgG2 to the bacteria. Our results show that protective immunity against Gram-negative bacterial pathogens can be elicited, and infectious diseases caused by these bacteria can be prevented by removing natural anti-αGal antibodies.

## 1 Introduction

The role of antibodies in host defense against infection by numerous microbes is undeniable. However, in some circumstances, antibodies may enhance the infective potential of microbes within the host. The clinical pathogenic effect of antibody-dependent enhancement (ADE) of infection is acknowledged in viral infections when a previous infection or vaccination leads to suboptimal non-neutralizing antibodies in serum against the infecting virus, facilitating its infectivity (1, 2). The precise mechanism of ADE in viral infections is not clearly understood. However, the most accepted pathway is the interaction of phagocytic cells bearing Fc receptors with virus-antibody immunocomplexes, facilitating the virus internalization and increasing infection (3).

The evidence for ADE of bacterial infections is less clear (4, 5). However, the existence of blocking or inhibitory antibodies lacking killing activity and interfering with bactericidal antibodies has been known for many years. ADE has been demonstrated in infections caused by *Pseudomonas aeruginosa* (6, 7), *Salmonella* spp (8)., uropathogenic *Escherichia coli* (9), and *Neisseria meningitidis* (10, 11). Antibodies responsible for ADE of bacterial infections may include natural anti-carbohydrate antibodies present before the immune system produces specific antibodies against pathogens. Thus, antibodies targeting poly-*N*-acetylglucosamine (PNAG) interfere with the protective antibodies induced by *Staphylococcus aureus* infection or vaccination (12). In addition, natural antibodies against Galα1-3Galβ1-4GlcNAcβ (αGal) epitope may act as blocking antibodies against Gram-negative bacteria (13). The antigenic stimulation by bacteria from normal gut microbiota may trigger the production of natural anti-αGal antibodies in humans (14, 15). However, anti-αGal antibodies demonstrated significantly greater binding to Gram-negative bacteria isolated from human blood and gallstones than to the same bacteria isolated from stool (13, 16). Furthermore, the binding of anti-αGal antibodies to blood-isolated bacteria impaired the complement-mediated killing of some pathogens (13).

Mammals express the αGal epitope except for apes, humans, and Old-World monkeys due to the inactivation of the gene coding for the α1,3-galactosyltransferase enzyme (17). Likewise, the lack of the epitope leads to the generation of natural anti-αGal antibodies by humans and non-human primates. These antibodies are involved in the initial rejection of xenografts or the long-term deterioration of mammal tissues exposing this structure (18, 19). In addition, like most anti-carbohydrate antibodies, these antibodies have a broad reactivity besides anti-αGal, binding to other related α-galactosyl residues and even with non-α-galactosyl-terminated oligosaccharides (20, 21). Thus, anti-αGal antibodies react with cells that do not express the αGal determinant, such as red blood cells of patients with β-thalassemia, sickle cell anemia, and normal senescent red blood cells or with ssDNA (22, 23). In addition, anti-αGal antibodies bind several microorganisms besides Gram-negative bacteria, including Gram-positive bacteria, viruses, and protozoa (24–26).

We hypothesized that removing anti-αGal antibodies might improve the killing of Gram-negative bacteria. Previous work showed that a soluble polylysine conjugate of αGal (GAS914) efficiently binds *in vivo* to circulating αGal xenoantibodies, leading to the intracorporeal removal of these antibodies in primates without side effects (27). Here, we assess whether the depletion of anti-αGal antibodies with GAS914 boosts the immune responses against Gram-negative bacteria.

## 2 Materials and methods

### 2.1 α1,3-galactosyltransferase knockout mice

Animal studies were performed in α1,3-galactosyltransferase knockout (GalT-KO) mice. All animal procedures were supervised and approved by Bellvitge Biomedical Research Institute (IDIBELL) ethics committee for animal experimentation and the Catalonia Government (DMA 3225). The care and handling of the animals conformed to the Guide for the Care and Use of Laboratory Animals published by the US National Institutes of Health (NIH Publication 85-23, revised 1996) and the European Agreement of Vertebrate Animal Protection for Experimental Use (86/609). Mice were maintained in the AAALAC-certified animal facility of IDIBELL under controlled temperature (21 ± 1°C), humidity (55 ± 5%), and cycles of light/dark (12/12 h), and food and water were given *ad libitum*. We used 16-week-old GalT-KO mice for all procedures, and each experimental group contained an equal number of male and female mice. Mouse blood collection was performed without anesthesia by submandibular bleeding. Serum was collected by mild centrifugation and stored at -20°C until further analysis.

The euthanasia procedure was established following the European Directive on protecting animals used for scientific purposes (2010/63/EU) and was performed in a CO_2_ chamber. Animal death was never considered an endpoint criterion. Instead, this was determined through a protocol of animal observation with the corresponding corrective measures (**Supplementary Table S1**).

### 2.2 GAS914

GAS914 (Novartis Pharma AG, Basel, Switzerland) is a poly-L-lysine backbone with an average degree of polymerization of 1,000 L-lysines and with 23-28% of lysines derivatized with αGal (27).

### 2.3 Quantification of anti-αGal antibodies by ELISA

The quantification of anti-αGal antibodies by ELISA followed the general protocol previously described (15).

### 2.4 Glycan array analysis

Glycochips (microchips printed with 577 different carbohydrate structures) were printed by Semiotik LLC (Moscow, Russia) using a collection of amine-functionalized glycans and bacterial polysaccharides covalently coupled to N-hydroxysuccinimide-derivatized glass slides (slide H, Schott-Nexterion, Mainz, Germany). The step-by-step protocol was deposited in Protocol Exchange (28). All data analysis was performed with the ScanArray^®^ Express Microarray Analysis System (PerkinElmer, Waltham, MA, USA). The binding results were expressed in relative fluorescence units (RFU) as median ± median absolute deviation (MAD). Interactive exploration of multidimensional data (heat mapping and clustering analysis) was performed with the Hierarchical Clustering Explorer application developed by the University of Maryland, MD, USA (http://www.cs.umd.edu/hcil/hce).

### 2.5 Cecal ligation and puncture procedure

The Cecal ligation and puncture (CLP) procedure was performed as described elsewhere (29). GalT-KO mice were deeply anesthetized with 4% isoflurane (Esteve Veterinaria, Barcelona, Spain), and during the procedure, anesthesia was maintained at 2.5% isoflurane. Under sterile conditions, a 1-2 cm midline incision was made, and the cecum was exteriorized and ligated (4-0 Safil^®^ Violet, B. Braun, Melsungen, Hessen, Germany) distally to the ileocecal valve. To generate moderate-grade sepsis, defined as ~ 15 - 50% mortality during the acute phase of sepsis, 25% of the cecum (~ 0.5 cm) was ligated and punctured twice with a 30-gauge needle. The abdominal wall incision was closed, and recovery was facilitated by keeping the animal on a thermal blanket. Buprenorphine (0.05 mg/kg) and meloxicam (1 mg/kg) were administered subcutaneously (sc) as analgesics before and after the CLP procedure. The animals were returned to their cages one hour after surgery, where water and food were provided *ad libitum* and monitored twice a day for 15 days, to asses both early and chronic sepsis mortality (30). Animal recovery was also favored by sc of 1 mL dextrose solution administration for up to 7 days. Access to water and food was facilitated two hours after surgery when animals were placed in their corresponding cages. Body weight, mobility, food intake, cutaneous features, and respiratory frequency of animals were monitored twice a day over 15 days, providing a score that led to different corrective measures depending on animal status (**Table S1**) (31).

### 2.6 Blood bacterial characterization after the CLP procedure

GalT-KO mice treated with 10 mg/kg of GAS914 or PBS sc on days -1, -3, and -5 before CLP (day 0) and scores ≥ 9 were euthanized at 12 h after the procedure (**Table S1**). Animals with scores < 9 were euthanized at 24 h after the CLP. To characterize bacteremia in peripheral blood, 100 µL of whole blood was seeded on a Petri dish with Blood Agar (Sigma-Aldrich, St. Louis, MO, USA) and incubated at 37°C for 24 h. Subsequently, bacteria were identified by a combination of different methods: Gram staining, plate culture with McConkey Agar differential medium, and biochemical tests (catalase and coagulase).

### 2.7 Stratification of bacterial DNA by pulsed-field gel electrophoresis

The DNA of the *E. coli* isolated from blood cultures obtained from GalT-KO mice after the CLP procedure was stratified by pulsed-field gel electrophoresis (PFGE). Ten colony-forming units or more were randomly selected from blood agar plates, resuspended in 5 ml of tryptic soy broth (Becton Dickinson and Company, Franklin Lakes, NJ, USA), and incubated at 37°C overnight. Total DNA was extracted following standard procedures (32). DNA restriction of each isolate was carried out with 40 units of *Xba*I (New England BioLabs Inc., Beverly, MA, USA) in 40 µL of 1xNEB2 buffer for 6 h at 37°C. The chromosomal DNA fragments were separated in a 1% agarose gel (CertifiedTM Megabase Agarose, Bio-Rad, Hercules, California, USA) in 0.5X TBE mM separated from the values: TBE (890 mM Tris; 890 mM boric acid; 20 mM EDTA, pH8). The pulsed-field gel electrophoresis (PFGE) was carried out in a CHEF-DRIII apparatus (Bio-Rad, Hercules, California, USA) with 0.5X TBE as running buffer. The Lambda Ladder PFGE Marker (New England Biolabs Inc., Beverly, MA, USA) was used as molecular weight marker. The running conditions were: 6V/cm with initial pulses of 1 second, which increased until 30 seconds, for a total run of 18 hours. PFGE patterns were visually compared following the criteria previously described (33). The dendrogram showing the clustering of strains was generated from the analysis of PFGE profiles with the FINGERPRINTING II software, with 1% optimization and band position tolerance. The cut-off value to define the PFGE patterns was set at 80%.

### 2.8 Bacterial strains, culture conditions

Two different strains of *Escherichia coli* were used in the studies: the human pathogenic *E. coli* O86:B7, obtained from the American Type Culture Collection (ATCC^®^ 12701™, LGC Standards, Teddington, Middlesex, UK), and one representative strain isolated from GalT-KO mouse blood after CLP (*E. coli* A3). In addition, *Pseudomonas aeruginosa* (strain 21565) and *Klebsiella pneumoniae* (strain 35204) were isolated from sputum and human blood, respectively. Both strains belonged to the Microbiology Department of Bellvitge University Hospital collection, were characterized as multidrug-resistant bacteria and are representative of bacteria causing often infectious diseases in the hospital. The strains were cultured for 16 h in Nutrient Broth (NB) medium (Becton Dickinson, and Company, Franklin Lakes, NJ, USA) at 37°C and 200 rpm (New Brunswick Scientific, Edison, NJ, USA). In the case of solid cultures, NB was supplemented with 1.5 (w/v) agar and incubated under similar conditions. The overnight cultures were diluted (1:100) with fresh NB medium for strain conservation. Cells were grown under the same conditions until the exponential phase (between 2-3 hours), where cellular suspensions were supplemented with 15% (v/v) glycerol (Sigma-Aldrich, St. Louis, MO, USA) and stored at -80°C for up to 2 years.

### 2.9 Bactericidal assay

Bacterial strains were incubated overnight in NB medium on an orbital shaker (New Brunswick Scientific, Edison, NJ, USA) at 200 rpm and 37°C. The bacterial suspension was diluted 100 times using a fresh medium the next day. Sterile baby rabbit complement (AbD Serotec, Kidlington, Oxfordshire, UK) was added to the bacterial suspension (2.5%, v/v). This mix was then incubated with the heat-inactivated (0.5 h at 56°C) mouse or human serum samples (5%, v/v) for 2-4 h at 200 rpm and 37°C. Cultures on 1.5% (w/v) agar NB plates were plated hourly (40 µL) at adequate dilutions (starting from 1:3125). After 18 h of incubation at 37°C, the resulting bacterial colonies were counted. The broth alone was used as the control for bacterial growth, and complement without serum was used for lysis via the alternative complement pathway (34). We calculated the bactericidal activity, comparing the number of bacteria in reaction mixtures containing the tested serum with the control.

### 2.10 Opsonic killing of *E. coli* O86:B7

Phagocytosis assay was performed based on a protocol described previously (35). Briefly, RAW 264.7 mouse macrophages were incubated in Dulbecco’s Modified Eagle Medium (DMEM) with a 5% Fetal Bovine Serum (FBS) at 37°C, 5% CO_2_, and 4.2 x 10^5^ cells were placed in 6-well cell culture plates. Meanwhile, *E. coli* O86:B7 was incubated overnight in NB medium on an orbital shaker (New Brunswick Scientific, Edison, NJ, USA) at 200 rpm and 37°C.

The next day, the bacterial suspension was diluted 100 times using a fresh medium and incubated in the same conditions until the exponential phase. Later, bacterial suspension was centrifuged (4,000*g*, 10 min, 22°C), and cell concentration was adjusted to 5 x 10^8^ CFU/mL. Then, bacteria were stained with 20µM of the SYTO9 Green marker (Thermo Fisher Scientific, Waltham, MA, USA), and 10^7^ stained bacteria were pre-opsonized with heat-inactivated human serum at 10% (v/v) in PBS, previously treated overnight at 4°C with GAS914 (100 μg/ml) by a 20 min incubation at RT in orbital shaking). Meanwhile, macrophages were incubated (20 min, in the dark, without agitation, at room temperature) with 2.5 μg/mL of APC-anti-mouse-F4/80 (Thermo Fisher Scientific, Waltham, MA, USA).

After a washing step, stained bacteria and macrophages were incubated (multiplicity of infection (MOI) of 10) at 37°C, 5% CO_2,_ for 30 minutes. After a washing step, the samples were fixed in 400 µL of paraformaldehyde (4% v/v), and cytofluorometric determinations were performed on a Gallios flow cytometer (Becton, Dickinson and Company, Franklin Lakes, NJ, USA). Every single FACS determination recorded about 20,000 total events. The events characterized by normal forward scatter (FSC) and side scatter (SSC) parameters were included in subsequent analyses. Fluorescence was collected through the corresponding bandpass filters for each indicated surface cell marker. Data were analyzed using KALUZA software (Beckman Coulter, CA, USA).

### 2.11 Antibody and complement deposition on *E. coli*

*E. coli* strains were incubated overnight in NB medium at 37°C with shaking at 200 rpm. The next day, the bacterial suspensions were diluted 100 times using a fresh medium and grown to an optical density of 0.3 units at 600 nm. Cells were harvested at 4,000*g* for 10 min at 4°C (Sorvall, Thermo Fisher Scientific, Waltham, MA, USA) and washed twice with an equal volume of Hank’s Balanced Salt Solution (HBSS, Life Technologies, Carlsbad, CA, USA). Cells were resuspended in an equal volume of HBSS supplemented with 1.3 mM calcium and 0.8 mM magnesium, divided (150 µL) into 96-well plates (conic bottom), and harvested at 2,500*g* for 10 min at 4°C (Eppendorf, Hamburg, Germany). Supernatants were carefully removed by aspiration. Heat-inactivated serum was added to the bacterial suspension at 10% (v/v) in supplemented HBSS. The cells were homogenized, and, in the case of complement deposition assays, 10% (v/v) standard mouse or human complement (Sigma-Aldrich, St. Louis, MO, USA) was added to the cell suspension. Plates were incubated for 0.5 h at 25°C and centrifuged under the same conditions after adding 100 µL of HBSS for washing. Supernatants were discarded by aspiration. Cells were then incubated in darkness (0.5 h at 4°C) with the secondary antibody labeled with the corresponding fluorochrome: anti-mouse C3 and C4 (Cedarlane, Burlington, ON, Canada), anti-human C3 and C4 (MP Biomedicals, Santa Ana, CA, USA), anti-mouse IgM, IgG (Invitrogen, Carlsbad, CA, USA), and IgG subclasses (Abcam, Milton, Cambridge, UK), and anti-human IgM, IgG, IgA (Invitrogen, Carlsbad, CA, USA), and IgG subclasses (Sigma-Aldrich, St. Louis, MO, USA), following the manufacturer’s instructions. After adding HBSS (100 µL) for washing, plates were centrifuged under the same conditions, and supernatants were discarded by aspiration. Finally, the cells were fixed (1 h at 4°C) with 4% (w/v) paraformaldehyde in HBSS.

Cytofluorometric determinations were performed on a Gallios flow cytometer (Becton Dickinson and Company, Franklin Lakes,NJ, USA). Every single FACS determination recorded about 20, 000 total events. The events characterized by normal forward scatter (FSC) and side scatter (SSC) parameters were included in subsequent analyses. Fluorescence was collected through the corresponding bandpass filters for each indicated surface cell marker. Data were analyzed using KALUZA software (Beckman Coulter, CA, USA). Cells incubated with supplemented HBSS alone were used as the negative control, and cells incubated with supplemented HBSS and secondary antibodies were considered the experiment’s background.

### 2.12 Lipopolysaccharide profiles

Bacterial strains were grown in NB medium for 16 h at 37°C (200 rpm). Cultures were adjusted to an optical density of 0.5 units at 600 nm with fresh medium, the cells were pelleted by centrifugation (10,600*g* for 10 min, Eppendorf, Hamburg, Germany), and the supernatants were discarded. Samples were prepared from whole-cell lysates treated with 0.5 mg/mL proteinase K (Sigma-Aldrich, St. Louis, MO, USA). Lipopolysaccharide (LPS) was extracted using the hot aqueous-phenol method previously described (36). Subsequently, the extract was separated by SDS-PAGE (13%) and directly stained using a standard silver stain protocol (Bio-Rad, Hercules, CA, USA). LPS from *E. coli* O111:B4 was used as a standard for long-chain O-antigen (Sigma-Aldrich, St. Louis, MO, USA).

### 2.13 αGal and α-Galactosyl antigen expression

Bacterial strains were incubated for 16 h in NB medium on a shaker at 200 rpm and 37°C. The next day, the bacterial suspensions were diluted 100 times using a fresh medium and grown to an optical density of 0.3 units at 600 nm. Cells were harvested (5,000*g*, 10 min at 4°C) and washed twice with an equal volume of HBSS. Next, cells were resuspended in an equal volume of HBSS, divided (150 µL) into 96-well plates (conical bottom), and centrifuged at 2,500*g* for 10 min at 4°C. Supernatants were carefully removed by aspiration. Cells were then incubated for 0.5 h at 4°C (in darkness) with 100 µL of lectin IB4-FITC in HBSS (1:100, EY Laboratories, San Mateo, CA, USA) for α-galactosyl expression (37). For specific αGal expression, anti-αGal IgM (Gal-13) monoclonal antibody (titer ~1:1000) and anti-αGal IgG (M86) monoclonal antibody (titer ~1:80) (kindly facilitated by Dr. Uri Galili) were used. After adding 100 µL of HBSS for washing, the plates were centrifuged again under the same conditions, and supernatants were removed by aspiration. Finally, the cells were fixed (1 h at 4°C) with 4% (w/v) paraformaldehyde in HBSS. Cytofluorometric determinations were performed on a Gallios flow cytometer (Becton, Dickinson and Company, Franklin Lakes, NJ, USA). Every single FACS determination recorded about 20, 000 total events. The events characterized by normal forward scatter (FSC) and side scatter (SSC) parameters were included in subsequent analyses. Fluorescence was collected through the corresponding bandpass filters for each indicated surface cell marker. Data were analyzed using KALUZA software (Beckman Coulter, CA, USA). Cells incubated with HBSS alone were used as the negative control for the experiment.

### 2.14 Statistical analysis

All experiments were performed at least three times unless otherwise stated. All data were analyzed using GraphPad Prism statistics software (GraphPad Software Inc., San Diego, CA, USA). First, the Gaussian data distribution was checked by the D’Agostino-Pearson omnibus normality test (alpha = 0.05), and the homogeneity of variances was determined by the F test (alpha = 0.05). Statistical analyses were performed using paired or unpaired parametric t-tests. The Wilcoxon matched-pairs signed-rank and Mann-Whitney test (unpaired data analysis) were used as non-parametric tests when data did not follow a Gaussian distribution. Grubbs’ test (extreme studentized deviation) was used to determine significant outliers in the data (alpha = 0.05). The results are expressed as the mean ± standard deviation (SD) and, in the case of glycan arrays, as the median ± median absolute deviation (MAD). Survival analysis was done with the Mantel-Cox log-rank test. Differences were considered statistically significant when p < 0.05.

## 3 Results

### 3.1 Removal of circulating anti-αGal antibodies in α1,3-galactosyltransferase knockout mice with GAS914

**Figure 1:**
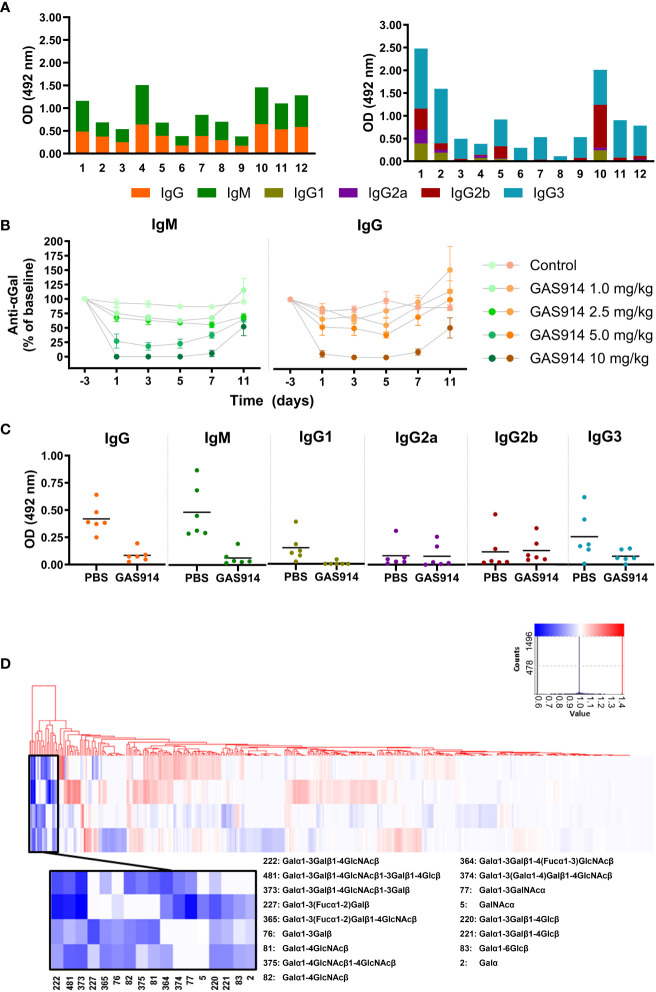
GAS914 removes most circulating anti-αGal antibodies in GalT-KO mice. **(A)** IgM and IgG anti-αGal antibody levels and the different IgG subclasses were measured by ELISA and expressed as relative optical density units at 492 nm. Each histogram represents the mean of three replicates (n = 12). **(B)** Pharmacodynamic studies. GalT-KO mice were subcutaneously injected with GAS914 (1.0, 2.5, 5.0, or 10 mg/kg) or PBS (control group) every other day (days 0, 2, and 4). Data are represented as the mean ± SD of four experiments. **(C)** Anti-αGal antibody removal by *in vivo* treatment with 10 mg/kg GAS914 or PBS (control group) every other day (days 0, 2, and 4). GalT-KO serum samples were obtained on day 5. IgM and IgG anti-αGal antibody levels and the different IgG subclasses were measured by ELISA and expressed as relative optical density units at 492 nm (n = 6). Data were analyzed by unpaired non-parametric Mann-Whitney test, *P < 0.05, **P < 0.01. **(D)** Pattern of anti-carbohydrate antibodies in GalT-KO mice treated with GAS914. Baseline and treated sera were assessed by glycan array technology using microchips printed with 577 different carbohydrate structures. All glycans were printed in 6 replicates. The heat map represents the ratio between the signal (relative fluorescence units) obtained for the serum after treatment with GAS914 and in baseline conditions. The selected clusters from the clustering analysis (amplified area) show the group of glycans for which the signal decreased (blue) or remained unchanged (white) due to the treatment with GAS914 (n = 4).

GalT-KO mice reproduce the natural concentration of anti-αGal antibodies observed in humans (38). All the mice in these studies exhibited high levels of anti-αGal antibodies without external immunization at 16 weeks (**Figure 1A**). The concentration of anti-αGal IgM and IgG were similar, and IgG3 was the predominant anti-αGal IgG subclass in most of the mouse serum samples (**Figure 1A**). No levels of anti-αGal IgA were detected in any animal. In addition, no anti-αGal antibodies were detected in wild-type (CBA) mice.

Mice were injected subcutaneously (sc) with 1, 2.5, 5, and 10 mg/kg of GAS914 on days 0, 2, and 4 (for three total doses). The best results were obtained with 10 mg/kg GAS914 (**Figure 1B**), which resulted in a decrease of approximately 90% in the level of circulating anti-αGal antibodies at the end of the challenge. With this dose, on day 11, one week after the last administration of GAS914, the levels of anti-αGal IgM and IgG antibodies were still 50% lower than those before the treatment (**Figure 1B**). The decrease in circulating anti-αGal antibodies was readily achieved with the first dose and changed very little with the two additional injections. Moreover, GAS914 significantly reduced both IgM and IgG, including the circulating anti-αGal IgG1 and IgG3 subclasses in GalT-KO mice (**Figure 1C**). No change occurred in IgG2a and IgG2b. Additionally, we did not observe any other side effects in the animals induced by the GAS914 treatment (**Table S1**).

The impact of the treatment with GAS914 on the specificity of natural mouse anti-glycan antibodies was assessed in four GalT-KO mice using microchips printed with 577 different carbohydrate structures and bacterial antigens. The glycochip analysis showed that GAS914 treatment significantly diminished antibody reactivity in all the mice against αGal trisaccharide (#222), αGal tetrasaccharide (#373), and αGal pentasaccharide (#481) structures. The treatment also significantly decreased antibody binding in some animals to other αGal and α-galactosyl residues (**Figure 1D**).

### 3.2 GAS914 improves survival after CLP in GalT-KO mice

**Figure 2:**
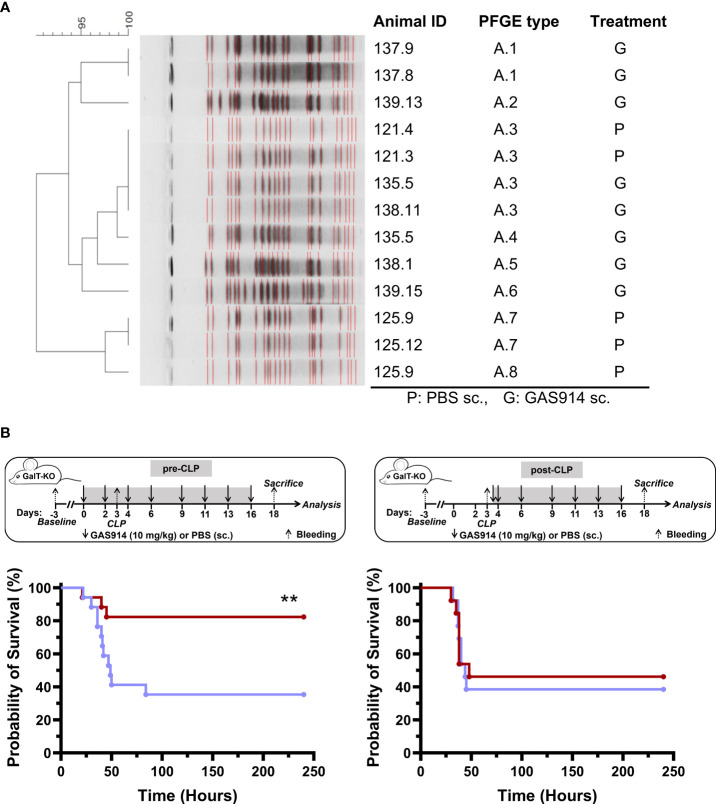
Treatment with GAS914 impacts GalT-KO mice survival after CLP. **(A)** DNA stratification by pulsed-field gel electrophoresis (PFGE) of *E coli* isolated from mouse blood after 12 h – 24 h of CLP in GalT-KO mice treated with GAS914 (n = 8) or PBS (n = 8). **(B)** Influence of GAS914 or PBS treatment on GalT-KO mouse survival after CLP in animals treated before and after the procedure (left, n = 17) or beginning the treatment 12 h after CLP (right, n = 14). Data are represented in Kaplan-Maier curves and were compared using the long-rank (Mantel-Cox) test, **P < 0.01.

The effect of removing anti-αGal antibodies *in vivo* was studied in GalT-KO mice submitted to CLP, which initially produces polymicrobial sepsis (2-12 h), shifting to predominant Gram-negative sepsis with coliform bacteria in the bloodstream at 24 h that causes, in most cases, animal death (39). Firstly, GalT-KO mice were pre-treated with GAS914 or PBS on days 0 and 2, after which CLP was produced on day 3. Then, animals were euthanized 12 or 24 h after CLP, depending on the welfare status (**Table S1**), to assess the presence of bacteria in the blood. Four animals of the PBS group were euthanized 12 h after CLP, and two of the PBS and 8 of GAS914 at 24 h. *E. coli* was isolated in 7 out of the 8 mice treated with GAS914 and 4 out of the 8 that received PBS. Two animals that received PBS died within 12 h after CLP, and *Enterococcus faecium* was the only one isolated from a third animal. We did not detect any bacteria in the blood of two mice (one from each experimental group). The DNA of the isolated *E. coli* strains was stratified by pulsed-field gel electrophoresis (PFGE), showing two different bacterial clusters (**Figure 2A**). One cluster included five *E. coli* strains isolated from GalT-KO mice treated with GAS914 and PBS, and the other cluster involved two isolated from PBS-treated mice. Only *E. coli* A3 was isolated from both GAS914 and PBS-treated mice.

Next, we evaluated the impact of GAS914 treatment on the mortality rate by introducing the therapy to the mice before or after the CLP procedure (**Figure 2B**). The results showed that the survival of GalT-KO mice that received two doses (days 0 and 2) of GAS914 before CLP (day 3) and on days 4, 6, 9, 11, 13, and 16 after CLP was significantly higher (83%) than that of control mice treated only with PBS (35%). By contrast, no benefit was observed in mice in which treatment with GAS914 was initiated 12 h after CLP (**Figure 2B**). The progression and severity of the infection were also scored by detailed observation of all animals (**Table S1**). Mice treated with GAS914 before and after CLP showed better welfare, and the disease severity was significantly lower than in the control group (**Supplementary Figure S1**).

### 3.3 Treatment with GAS914 increases the bactericidal capacity of GalT-KO mouse serum against *E. coli*

**Figure 3:**
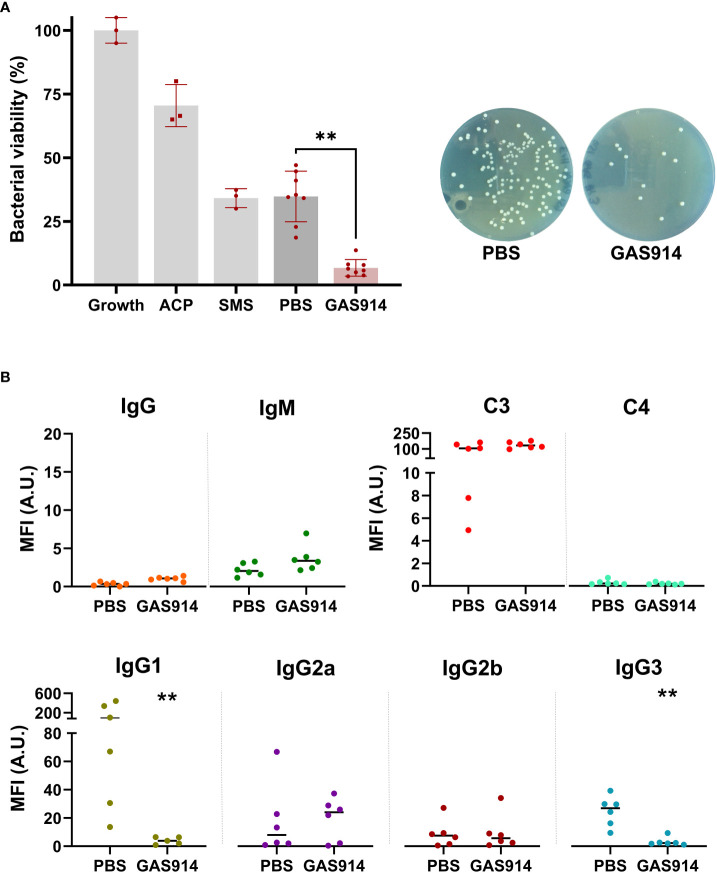
Treatment with GAS914 increases GalT-KO mice serum bactericidal capacity against *E coli* A3 isolated after CLP.**(A)** Effect of GAS914 and PBS in GalT-KO bactericidal activity against *E coli* A3. The bactericidal activity was calculated as the percentage of bacteria surviving in reaction mixtures containing the tested serum compared to the control (growth). Growth: control bacterial growth, ACP, alternative complement pathway; SMS, standard mouse serum. Individual data of PBS and GAS914 represent the mean of three experiments. **(B)** Median fluorescence intensity of IgG, IgM, C3, C4, and IgG subclasses on the surface of *E coli* A3 (n = 6). Individual data represents the mean of three experiments. Unpaired non-parametric Mann-Whitney test was used to compare PBS and GAS914 groups, **P < 0.01.

We also measured *in vitro* GalT-KO mice serum bactericidal activity, and the antibody and complement deposition against the pathogenic mouse isolate *E. coli* A3 and *E. coli* O86:B7 (ATCC^®^ 12701^™^), a bacterium with high α-galactosyl content (37, 40). The results showed an average 5.4-fold enhancement of the killing capacity of mice sera against *E. coli* A3 in animals receiving GAS914 compared to PBS treatment (**Figure 3A**). Furthermore, the increased serum bactericidal capacity with GAS914 treatment was associated with a significantly lower recognition of the bacterium by IgG1 and IgG3 subclasses in animals treated with GAS914 compared to those treated with PBS (**Figure 3B**). However, no changes were observed in total IgG, IgM, C3, C4, IgG2a, and IgG2b deposition. Interestingly, IgG1 showed substantially higher binding to *E. coli* A3 than the other IgG subclasses, and GAS914 reduced the reactivity of these antibodies by an average of 20-fold, despite the low concentration of this subclass among IgG anti-αGal antibodies (**Figure 1A**). In contrast, serum from GAS914-treated mice evidenced somewhat improvement in bactericidal activity against *E. coli* O86:B7 without changes in IgG subclasses deposition (**Figure S2**).

### 3.4 GAS914 boosts the bactericidal activity of human sera against human-infecting Gram-negative bacteria

The levels of anti-αGal antibodies in the serum of 8 healthy blood donors displayed a higher concentration of anti-αGal IgG than IgM and IgA antibodies and the predominance of the IgG2 subtype (**Figure S3**). GAS914 was combined with human sera from healthy blood donors *in vitro* at a 100 µg/mL concentration, estimated as the exposition achieved with *in vivo* doses of 5 mg/kg in primates (27). Exposure to GAS914 significantly reduced the detection of anti-αGal IgM, IgG, and IgA antibodies from individual human sera, along with the IgG2 subclass (**Figure S3**).

**Figure 4:**
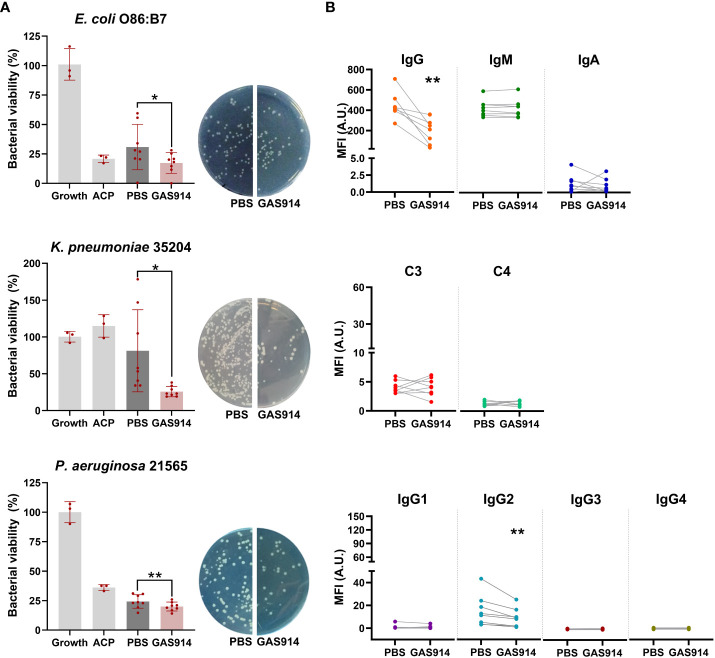
Neutralizing anti-αGal antibodies from human sera with GAS914 increases serum bactericidal killing and decreases IgG2 binding to Gram-negative bacteria. **(A)** Bactericidal killing after exposing the human sera to PBS or GAS914 against *E coli* O86:B7, *P. aeruginosa* 21565, and *K pneumonia* 35204 (n = 8). The bactericidal killing was calculated as the percentage of the number of bacteria surviving in reaction mixtures containing the tested serum compared to the control (growth). Growth: control bacterial growth, ACP, alternative complement pathway. Individual data of PBS and GAS914 represent the mean of three experiments. **(B)** Median fluorescence intensity of IgG, IgM, IgA, IgG subclasses, and complement C3 and C4 deposition on the surface of *E coli* O86:B7 (n = 8). Individual data represents the mean of three experiments. Comparisons were analyzed by paired *t*-tests, *P < 0.05, **P < 0.01.

Next, we investigated whether the blockade of anti-αGal antibodies from sera of human blood donors *in vitro* modifies the complement-mediated killing activity against human pathogenic Gram-negative bacteria isolates. These included human *E. coli* O86:B7 and multidrug-resistant (MDR) human *Pseudomonas aeruginosa* 21565 and *Klebsiella pneumoniae* 35204 isolates. Inhibition of anti-αGal antibodies with GAS914 *in vitro* increased an average of 2-fold the bactericidal activity of human sera against *E. coli* O86:B7, 3.5-fold against *K. pneumoniae* and 1.3-fold against *P. aeruginosa* 21565 (**Figure 4A**). Although *E. coli* strains are mainly killed in a bactericidal assay using antibodies and complement, they can also be killed by phagocytes after bacterial opsonization by antibodies (41). Therefore, we also tested the opsonophagocytic activity of macrophages against *E. coli* O86:B7. Blockade of anti-αGal antibodies with GAS914 in a pool with the 8 human sera was associated with augmenting bacterial phagocytosis by macrophages (**Figure S4**).

We subsequently tested the deposition of IgM, IgA, IgG, and IgG subclasses, C3, and C4, on the surface of *E. coli* O86:B7. Neutralizing anti-αGal antibodies with GAS914 from human sera was associated with a decreased binding of IgG, specifically IgG2 antibodies (**Figure 4B**). We also assessed the bactericidal activity, and antibody and complement deposition from the serum of healthy blood donors against the *E. coli* A3 strain (isolated from the blood of GalT-KO mice after CLP) after the *in vitro* inhibition of anti-αGal antibodies with GAS914. No changes occurred in the bactericidal capacity, antibody binding, or complement deposition in the serum from 8 healthy blood donors, regardless of whether anti-αGal antibodies were removed (**Figure S5**).

### 3.5 Lipopolysaccharide profile and αGal expression in Gram-negative bacteria

**Figure 5:**
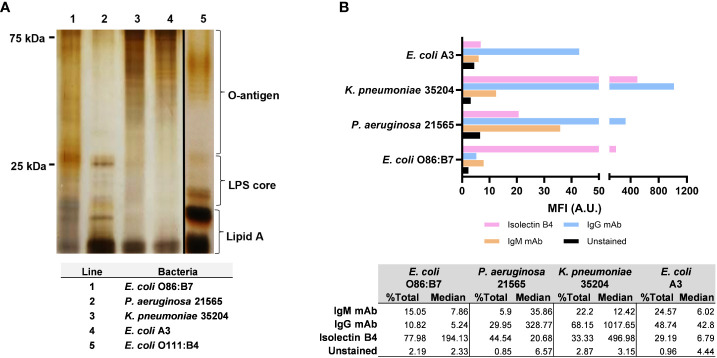
Length and expression level of lipopolysaccharide (LPS) O-antigen chains and αGal in Gram-negative bacteria. **(A)** Separation by SDS-PAGE (13%) of repeating oligosaccharide units of LPS extracted from *Escherichia coli* O86:B7 (lane 1), *Pseudomonas aeruginosa* 21565 (lane 2), *Klebsiella pneumoniae* 35204 (lane 3) and *Escherichia coli* A3 (lane 4). Standard LPS from *E coli* O111:B4 (lane 5, Sigma-Aldrich, St. Louis, MO, USA) was used as a reference. **(B)** Median fluorescence intensity of α-galactosyl expression with isolectin IB4 and αGal expression with anti-αGal IgM and IgG monoclonal antibodies in *E coli* A3, *K pneumoniae* 35204, *P. aeruginosa* 21565, and *E coli* O86:B7. Data are representative of three independent experiments.

It has been suggested that the impairment of serum killing by anti-αGal and other antibodies depends on binding antibodies to the long-chain O-antigen of LPS (10, 13), composed of many repeating oligosaccharide units. To characterize the Gram-negative bacteria used in the studies, we quantified and separated the repeating oligosaccharide units of LPS. We also assessed α-galactosyl expression in bacteria by lectin IB4-FITC staining and the presence of αGal by monoclonal antibodies. *E. coli* O86:B7 and *P. aeruginosa* 21565 did not show repeating oligosaccharides bands in the LPS O-antigen chains, indicating that both bacteria have short O-antigens of LPS. In contrast, *K. pneumoniae* 35204 and mouse *E. coli* A3 displayed repeating oligosaccharides bands in the LPS, like the pattern of a standard LPS with a long-chain O-antigen isolated from *E. coli* O111:B4 (Sigma-Aldrich, St. Louis, MO, USA) (**Figure 5A**).

The bacteria showed a variable expression of αGal and α-galactosyl residues assessed by monoclonal antibodies and IB4 lectin, respectively (**Figure 5B**). *K. pneumoniae* 35204 displayed the highest expression of αGal, followed by *P. aeruginosa* 21565 and *E. coli* A3. The expression of α-galactosyl residues was also very high in *K. pneumoniae* 35204, followed by *E. coli* O86:B7 and *P. aeruginosa* 21565. In *E. coli* O86:B7, there was no evidence of αGal residues, indicating that most of the antigen expressed in this bacterium corresponds to the B-blood group moiety (Galα1-3(Fucα1-2)Galβ) (40). In contrast, *E. coli* A3 showed some αGal without any α-galactosyl expression.

## 4 Discussion

To date, protective immunity without direct contact with an external microbe, the process of enhancing an individual immune system to prevent or mitigate infectious diseases, has been achieved by vaccination and the induction of antibodies with a killing capacity against the pathogen causing the infection. Here we describe for the first time a new approach to generate protective immunity: the removal of blocking or interfering anti-αGal antibodies involved in a mechanism of ADE of Gram-negative bacterial infections. Whether an antibody is protective or deleterious and causes ADE depends on several elements, including the expression of virulence factors by the bacteria, the titer of the antibody, and the particular class and subclass of antibodies reacting with the pathogen. The depletion of natural anti-αGal antibodies with GAS914 effectively overcame these factors and boosted the immune response against Gram-negative bacteria, including those resistant to multiple antibiotics.

Natural anti-αGal antibodies, like most natural anti-carbohydrate antibodies, develop from the stimulation of microbiota (15). Interestingly, as we previously showed, the levels of these antibodies evidenced substantial variations among genetically identical mice maintained under the same housing conditions (15). However, regardless of the concentration of anti-αGal antibodies, GAS914 removed both IgM and IgG, including IgG1 and IgG3 subtypes, reacting to the αGal moieties in all the GalT-KO mice. In contrast, antibodies that react with other alpha-galactosyl or non-alpha-galactosyl structures showed different patterns of change after the GAS914 treatment, varying from animal to animal.

The CLP model was originally developed to understand the natural history of untreated infection (42). We previously showed a significant reduction of anti-αGal antibodies 12-24 h after CLP in GalT-KO mice non-treated with GAS914, likely due to the binding of these antibodies to the infecting bacteria that are isolated in all the cases early after the procedure from the bloodstream (43, 44). The present study evidenced *E. coli* strains as the primary pathogen causing sepsis after CLP in GalT-KO mice and that removing anti-αGal antibodies with GAS914 boosted the killing of these pathogens, protecting animals from lethal infections. One of the elements associated with a deadly outcome of CLP is the early increase in plasma cytokines like IL-6 after the procedure (45). This augment was not observed in GalT-KO mice treated with PBS before CLP (43). However, treatment with GAS914 led to an augment of leptin, CXCL1, CXCL13, and TIMP-1 that have been associated with improved survival after CLP (46, 47). Another factor affecting the result of CLP is the existence of blocking antibodies before the procedure, which prevents bacterial killing and increases mortality (45). Our studies evidenced that removing anti-αGal antibodies before CLP improved bacterial killing and enhanced animal survival, confirming that these antibodies are blocking antibodies for Gram-negative bacteria. In contrast, initiating GAS914 treatment 12 h after the CLP procedure did not benefit GalT-KO mice. This suggests that the main impact of eliminating anti-αGal antibodies is prevention, not the treatment of infections. However, a robust immune response can reduce the duration of antibiotic therapy (48). Thus, it cannot entirely be ruled out that anti-αGal antibody elimination may also benefit the treatment of infectious diseases by Gram-negative bacteria by reducing the period of antibiotic therapy.

Long lipopolysaccharide (LPS) O-antigens are a leading factor of bacterial virulence and resistance to serum killing (49), contributing to the spread of multidrug-resistant bacteria and influencing the host response during sepsis (50, 51). The binding of antibodies to the O-antigens of the long LPS surface of Gram-negative bacteria instead of the capsular polysaccharide impairs bacterial killing (13). In this study, removing anti-αGal antibodies with GAS914 markedly increased the complement-mediated killing of *E. coli* A3 and *K. pneumonia* 35204 displaying long LPS O-antigens. However, it also improved, although to a lesser extent, the killing of *E. coli* O86:B7 and *P. aeruginosa* 21565, bacteria with short LPS O-antigens. Along with the site to which the antibodies attach, another phenomenon relevant to developing ADE of bacterial infections is the IgG subclass that binds to the bacteria. Reactivity mediated by human IgG2 antibodies impaired the killing of bacterial pathogens, whereas the binding of IgG1 did not (7, 9). In our study, removing anti-αGal antibodies in GalT-KO mice almost entirely killed *E. coli* A3 by significantly reducing the binding of mouse IgG1 and IgG3 to the pathogen. In the case of *E. coli* O86:B7, which expresses other α-galactosyl residues different from αGal, eliminating anti-αGal antibodies from human serum reduced the binding of IgG2 antibodies to the bacteria. IgG3 antibody responses in mice and IgG2 in humans are mainly directed to carbohydrate antigens, whereas the equivalent of mouse IgG1 in humans is IgG4, which reacts to infectious and non-infectious antigens (52). All these subclasses of IgG have in common that they have a limited ability to mediate complement and/or cell-mediated killing (53). On the other hand, the binding of these antibodies to bacterial antigens is independent of the overall concentration of IgG subclasses (7), as we showed with mice IgG1 anti-αGal subclass.

The impact on bactericidal activity and serum antibody reactivity from GAS914 mouse-treated animals against *E. coli* O86:B7 was much lower than that observed against *E. coli* A3. Similarly, removing anti-αGal antibodies from human serum did not affect the killing and human antibody binding to mouse *E. coli* A3. These different effects of removing anti-αGal antibodies from humans and mice may reflect the host specificity of many bacteria, with tropism for particular species and specific individuals within one species (7, 54). Along with the molecular interactions between the pathogen and host (54), existing antibodies like αGal also appear to play an essential role in shaping the composition of intestinal bacteria (55).

Current antibody-removal therapies are extracorporeal techniques like plasma exchange by plasmapheresis, immunoadsorption through columns containing protein A to eliminate IgG, or immunoadsorption through columns carrying blood ABO epitopes that adsorbs anti-ABO antibodies (56). Also, the IgG-degrading enzyme derived from *Streptococcus pyogenes* (Imlifidase) is a novel agent that cleaves all four human subclasses of IgG and has been used for anti-HLA antibody desensitization in kidney transplantation (57). Plasmapheresis has been used to remove IgG2 antibodies in patients with bronchiectasis and chronic *P. aeruginosa* infections, improving their clinical conditions (58). An immunoadsorption technique with columns carrying multiple αGal oligosaccharides was also used to remove circulating anti-αGal antibodies to prevent early xenograft rejection in primates (59). All these extracorporeal procedures, and also Imlifidase, effectively eliminate specific or non-specific antibodies but require the combination of immunosuppression to prevent the reappearance of antibodies. This restrains extracorporeal techniques to remove antibodies in autoimmune diseases or transplantations that also need immunosuppressive treatments. In contrast, GAS914 binds to circulating anti-αGal antibodies leading to the formation of immune complexes that are quickly metabolized by the liver and excreted by the kidney without an immune response against either the carbohydrate or the backbone (27). As was previously shown in non-human primates (27), the first dose of GAS914 cleared most of the circulating anti-αGal antibodies in GalT-KO mice. Besides removing circulating anti-αGal antibodies, GAS914 lowers the production of new antibodies without needing other immunosuppressive treatments. This may result from the accumulation of GAS914 in the lymphoid organs, in the area of B cells (27), which may inhibit antibody production or locally absorb newly formed antibodies preventing them from reaching circulation.

The immediate effect of GAS914 for removing anti-αGal antibodies correlates with the prompt generation of immunity against Gram-negative pathogens. This provides a substantial advantage to removing anti-αGal antibodies compared to producing new antibodies by traditional vaccines, for instance, to prevent hospital-acquired infections. For example, a vaccine developed to avoid *P. aeruginosa* infections in patients admitted to ICU showed the production of specific IgG antibody titers at day 14 after immunization (60). However, it failed to prevent *P. aeruginosa* infection and disease, likely because most occurred before seroconversion on day 14. Other groups that might benefit from removing anti-αGal antibodies are neutropenic patients and those undergoing dialysis therapies (61, 62). In both cases, higher serum levels of anti-αGal antibodies were associated with a greater incidence of infectious diseases.

Although the results presented here are promising, they have several limitations. First, the GalT-KO CLP model only isolated *E. coli* strains. Therefore, we cannot rule out that improving bactericidal activity by removing anti-αGal antibodies is distinct from other Gram-negative mice bacteria. This limitation is somewhat mitigated by the results obtained with human serum samples against various pathogens. However, the bacteria used in those experiments did not cause infections in the individuals that provided the sera. Second, disparities in the clinical impact of anti-αGal antibodies have been described in human pneumococcal (24), fungal (63), protozoan (26, 64, 65) and viral infections (66). The titer of antibodies appears as one key element for the neutralization/killing of pathogens or the enhancement of infection, although with an opposite effect depending on the target microbe. Thus, low titers of antibodies enhance viral infections and are bactericidal, whereas high titers neutralize viruses but prevent bacterial killing (1, 5). In the particular case of natural anti-αGal antibodies bind and drive phagocytosis of *Streptococcus pneumoniae* despite the bacteria not expressing αGal (24). Also, antibodies induced during *Trypanosoma cruzi* and *Plasmodium spp* infections reacting with αGal have lytic capacity against the protozoan parasites but with different specificities than natural anti-αGal antibodies (26, 64, 65). Consequently, the potential benefit of these antibodies should be considered, along with the blocking effect of natural anti-αGal IgG antibodies on Gram-negative bacteria reported here. In the case of *S. pneumonia*, we showed that GAS914 only removes antibodies binding to αGal and α-galactosyl residues, which are not essential parts of the bacteria epitopes (24). On the other hand, we also evidenced a reduced inhibitory effect of GAS914 over anti-αGal IgG1 and IgG3 in humans and IgG2a and IgG2b in mice, which react to protein antigens and are associated with induced antibodies, suggesting the potential preservation of these antibodies with the treatment. In addition, we have shown that distinct-sized polylysines, and different percentages of αGal attachment, modify the binding capacity of the glycopolymers to individual anti-αGal antibodies, suggesting the possibility of generating tailored molecules to remove specific isotypes (67).

In summary, removing anti-αGal antibodies with GAS914 improves serum bactericidal activity against Gram-negative bacteria by reducing the binding of mouse IgG1 and IgG3 and human IgG2 to pathogens. This provides a compelling argument for pursuing the clinical use of removing anti-αGal antibodies to prevent infections caused by these bacteria, which are increasingly resistant to most available antibiotics. If the favorable safety and efficacy profiles demonstrated for GAS914 in primates are confirmed in humans (27), deleting anti-αGal antibodies may become a new therapy for preventing Gram-negative infectious diseases, particularly in healthcare settings.

## Data availability statement

The raw data supporting the conclusions of this article will be made available by the authors, without undue reservation.

## Ethics statement

The studies involving human participants were reviewed and approved by Clinical Research Committee of the Bellvitge University Hospital. The patients/participants provided their written informed consent to participate in this study. The animal study was reviewed and approved by Bellvitge Biomedical Research Institute (IDIBELL) ethics committee for animal experimentation and the Catalonia Government.

## Author contributions

SO-A, DB-G, and RM contributed to the conception and design of the study. MP-C and CC performed all the animal studies. SO-A, DB-G, and YF-A performed all the determinations of antibodies, bactericidal activity, binding of antibodies, and complement to the bacteria and LPS profile. MC and MD carried out the bacterial DNA stratification. JMV performed all the flow cytometries. NK, NS, and NB designed and prepared the glycan arrays. SO-A, DB-G, and RM wrote the manuscript. All the authors contributed to manuscript revision, read and approved the submitted version.
