# Supplementary material for: Removal of natural anti-αGal antibodies elicits protective immunity against Gram-negative bacterial infections
Source: Front Immunol. 2023 Aug 18;14:1232924. doi: 10.3389/fimmu.2023.1232924 (PMC10471972; doi:10.3389/fimmu.2023.1232924)
Supplement: Supplementary file 1 [file Presentation_1.pdf]

Supplementary Material

Removal of natural anti-αGal antibodies elicits protective immunity against Gram-negative bacterial infections

Olivera-Ardid, Daniel Bello-Gil, Magdiel Perez-Cruz, Cristina Costa, Mariana Camoez, M. Angeles Dominguez, Yara Ferrero-Alves, Jose Miguel Andres-Vaquero, Nailya Khasbiullina, Nadezhda V. Shilova, Nicolai V. Bovin, Rafael Mañez*

1 Supplementary Figures and Tables

1.1 Supplementary Table

Table S1. Protocol of observation of GalT-KO mice after the CLP procedure. Corrective measures applied to animals following the score of the observation protocol.

Parameter	Features	Score
Body weight loss	None	0
	< 10%	1
	Between 10% and 20%	2
	Between 10% and 20% and modification of the feces	3
	> 20%	4
	No food and water intake<br>(Immediately euthanized)	5
General appearance	Normal	0
	Dull and/or bristling coat	1
	Dull and/or bristling coat and/or ocular or nasal secretions	2
	Abnormal posture	3
Self-mutilation or signs of pain	Yes	3
	No	0
Response to stimulus	Normal	0
	Minor changes	1
	Reduced mobility and/or inactivity	2
	Aggressive or comatose, exhibiting convulsions, and/or tremors	3
**Score**	**Corrective measures**	
0 to 4	Direct observation (twice a day)	
5 to 8	Direct observation (twice a day) and buprenorphine administration (0.05 mg/kg/8 h)	
9 to 12	Direct observation (twice a day), buprenorphine administration (0.1 mg/kg/8 h), and euthanasia in the following 12 h	
>12	Immediate euthanasia	

1.2 Supplementary Figures

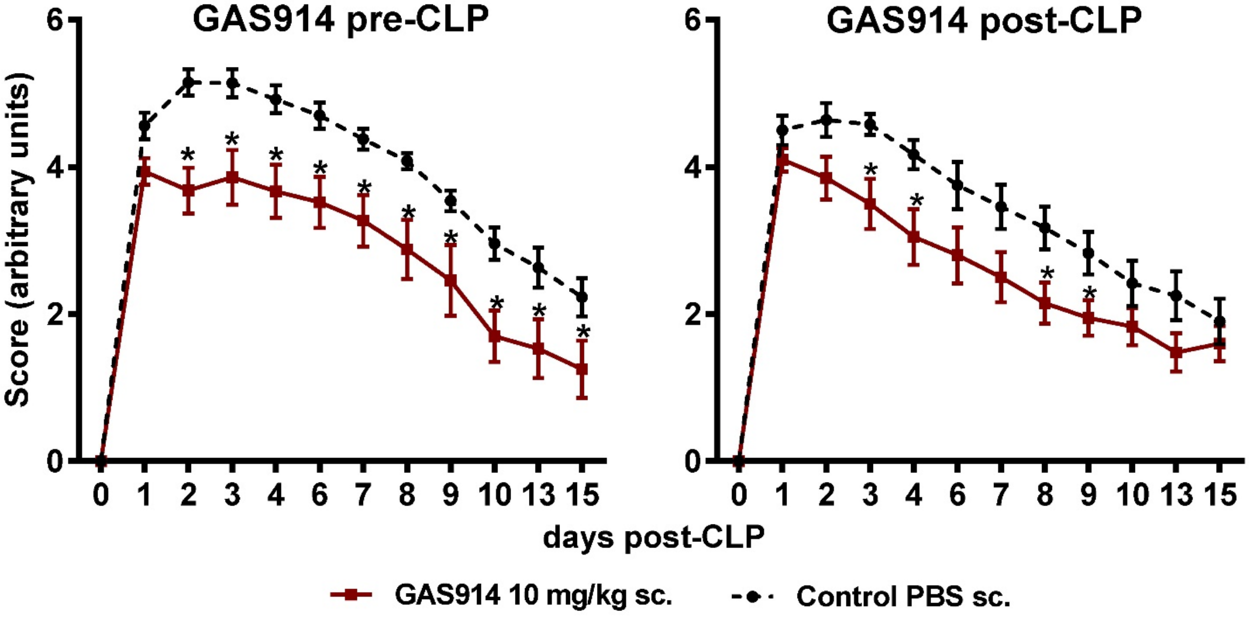

Figure S1. Influence of GAS914 treatment on overall GalT-KO mouse life quality after the cecal ligation and puncture (CLP) procedure. Each point represents the infection severity per day as a mean scoring (in arbitrary units), calculated following the observation protocol (n = 17). Error bars represent the standard deviation of these determinations (* $p < 0.05$).

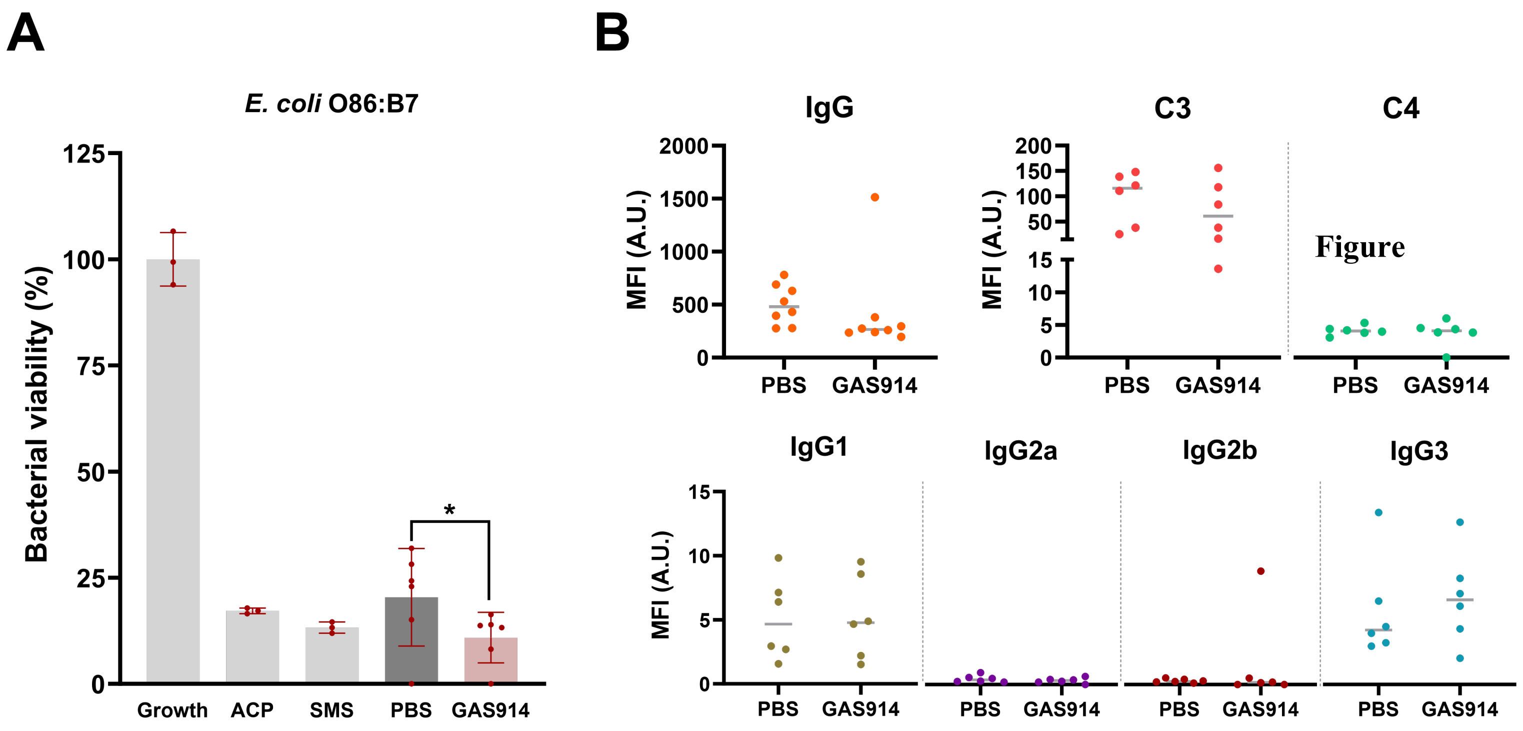

S2. Bactericidal activity of GalT-KO mice serum, and antibody and complement deposition against *E. coli* O86:B7. (**A**) Effect of PBS and GAS914 in GalT-KO bactericidal activity against *E. coli* O86:B7 representing the mean of three replicates. The bactericidal activity was calculated as the percentage of bacteria surviving in reaction mixtures containing the tested serum compared to the control (growth). Growth: control bacterial growth, ACP: alternative complement pathway, SMS: standard mouse serum. (**B**) Median fluorescence intensity of IgG, C3, C4, and IgG subclasses on the surface of *E. coli* O86:B7 (n = 6). IgM could not be performed for lack of sera. Individual data represents the mean of three experiments and were analysed by unpaired non-parametric Mann-Whitney test to compare PBS and GAS914 groups, $*P < 0.05$.

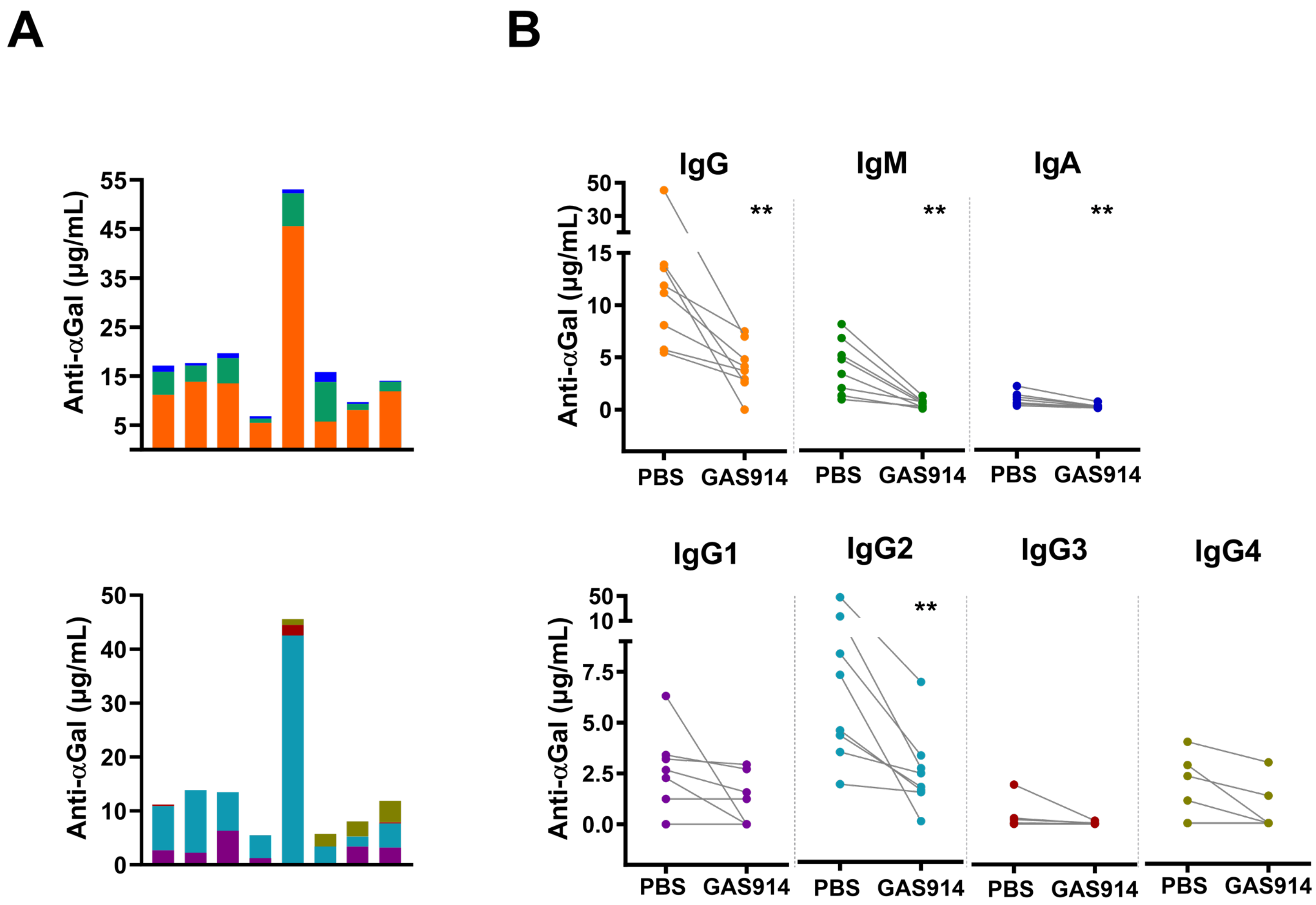

Figure S3. ***In vitro* inhibition of anti-αGal antibodies in human sera with GAS914.** (**A**) IgM, IgG, IgA, and IgG subclasses of anti-αGal antibody levels in 8 human sera measured by ELISA and expressed as μg/mL. Each coloured bar represents the mean of three replicates. (**B**) IgM, IgG, IgA, and IgG subclasses of anti-αGal antibody levels in 8 human sera exposed to PBS or 100 μg/mL of GAS914 expressed as μg/mL. Individual data represents the mean of three experiments, and the comparisons were analysed by paired *t*-tests, *$P < 0.05$.

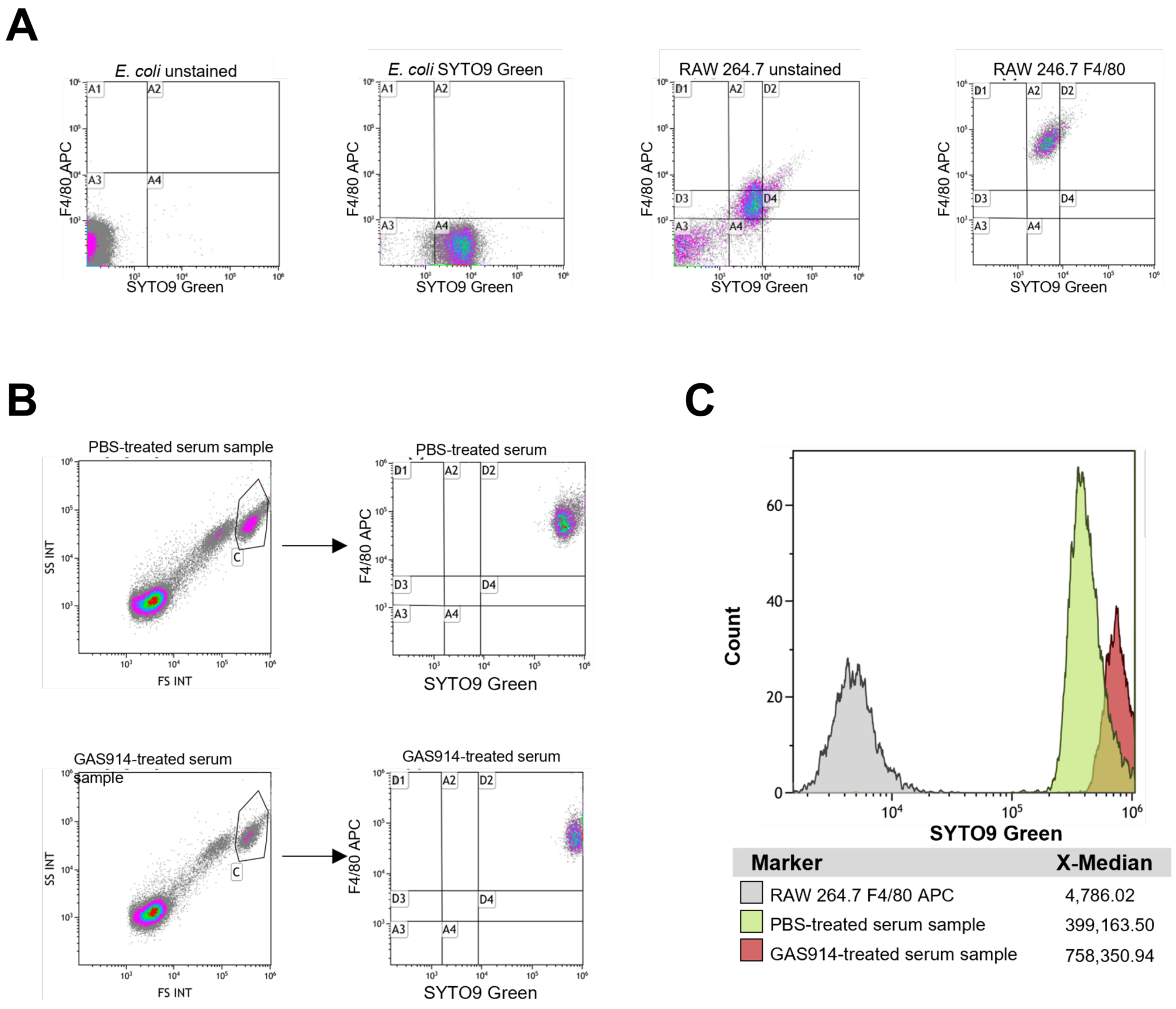

Figure S4. Macrophages RAW 264.7 phagocyte in higher rates *E. coli* O86:B7 preopsonized with GAS914-treated serum. (**A**): Negative controls of macrophages and E.coli O86:B7. (**B**): Representative example of gating strategy to define macrophage population. Events collected were displayed in a FS intensity (FS INT) vs. side scatter intensity (SS INT) plot to discard debris and define a total macrophage population. SYTO9 Green was added to the samples to detect the bacteria phagocyted by the macrophages. Anti-F4/80 antibody was used to detect the mouse glycoprotein, which is expressed at high levels on the cell surface. (**C**): Histogram showing the median of fluorescence intensity for macrophage population of a representative sample of each experimental group. F4/80-stained macrophages are plotted as a negative control of the background fluorescence in the absence of SYTO9 Green bacteria.

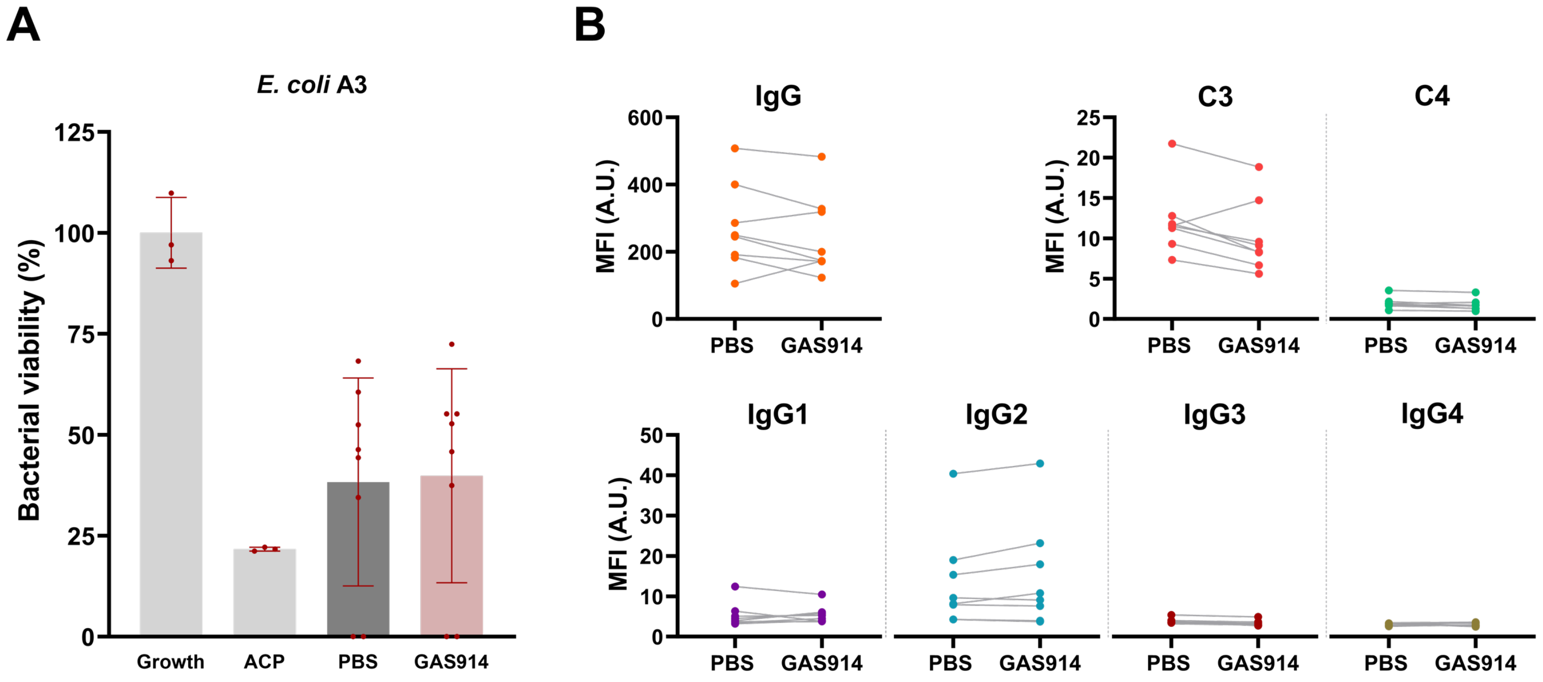

Figure S5. Bactericidal activity of human serum, and antibody and complement deposition against mouse *E. coli* 1. (**A**). Effect of PBS and GAS914 in human serum activity against mouse *E. coli* A3 (n = 8). The bactericidal activity was calculated as the percentage of bacteria surviving in reaction mixtures containing the tested serum compared to the control (growth). Growth: control bacterial growth, ACP: alternative complement pathway. (**B**) Median fluorescence intensity of human IgG, C3, C4, and IgG subclasses on the surface of mouse *E. coli* A3 (n = 7). IgM could not be performed for lack of sera. Individual data represents the mean of three experiments, and the comparisons were analysed by paired *t*-tests.
